# Supplementary material for: Enhanced production of taxadiene in Saccharomyces cerevisiae
Source: Microb Cell Fact. 2020 Nov 2;19:200. doi: 10.1186/s12934-020-01458-2 (PMC7607689; doi:10.1186/s12934-020-01458-2)
Supplement: Supplementary file 1 — Additional file 1: Table S1. Plasmids used in this study. Table S2. List of primers used in the study. Table S3. DNA sequences. Figure S1. Fluorescence imaging of GFP-tagged TASY showing spotted subcellular localization consistent with poor TASY solubility. Figure S2. Mass spectra for compounds produced by LRS5. Figure S3. LRS5 gas chromatogram showing additional potential terpenoids. Figure S4. Mass spectra of the LRS5 additional potential terpenoids (A-D) as shown in Figure S3. [file 12934_2020_1458_MOESM1_ESM.pdf]

Additional File 1

Table S1. **Plasmids used in this study**

Plasmid name	Description
pLR1	Yep_pGAL1-3Xflag-M60TASYBRE-tCYC1
pLR2	Yep_pSV1-3Xflag-M60TASY-tCYC1
pLR3	Yep_pVAN1-3Xflag-M60TASYBRE-tCYC1
pLR4	Yep_pADH1-3Xflag-M60TASYBRE-tCYC1
pLR5	Yep_pTDH3-3Xflag-M60TASYBRE-tCYC1
pL10	Yep_pGAL1-M60TASYBRE-tCYC1
pLR24	Yep_URA17_pGAL1-3Xflag-M60TASYBRE-tCYC1
pLR25	Yep_Leu2d_pGAL1-3Xflag-M60TASYBRE-tCYC1
pLR33	Yep_pTDH3-M60TASYBACA-tCYC1
pLR34	Yep_pTDH3-M50TTASYBRE-tCYC1
pLR35	Yep_pTDH3-KOZ-M50TTASYBRE-tCYC1
pLR36	Yep_pTDH3--M60TASYBRE-MBP-tADH1<br><br>pGAL1-T5aH-tCYC1
pLR37	Yep_pTDH3-CAS9-tADH1
pLR40	Yep_pGAL1-M40TASYBRE-tCYC1
pLR41	Yep_pGAL1-M44TASYBRE-tCYC1
pLR42	Yep_pGAL1-M48TASYBRE-tCYC1
pLR43	Yep_pGAL1-M52TASYBRE-tCYC1
pLR44	Yep_ pGAL1-M54TASYBRE-tCYC1
pLR45	Yep_ pGAL1-M56TASYBRE-tCYC1

pLR46	Yep_ pGAL1-M58TASYBRE-tCYC1
pLR47	Yep_ pGAL1-M62TASYBRE-tCYC1
pLR48	Yep_ pGAL1-M64TASYBRE-tCYC1
pLR49	Yep_ pGAL1-M66TASYBRE-tCYC1
pLR50	Yep_ pGAL1-M68TASYBRE-tCYC1
pLR51	Yep_ pGAL1-M70TASYBRE-tCYC1

Table S2. **List of primers used in the study**

**Name**	**Sequence**
M13 F Backbone Ups of Promoter	GTAAAACGACGGCCAGT
M13 R backbone down of Term	CAGGAAACAGCTATGAC
TASY1	GGTAGAGACATGTTGGCCCA
TASY2	AGCGGAGTATTCTGGTTCGA
TASY3	TCCGTCATTGCCTTGTCTGT
TASY4	ACCGATACCCAAATGTTCAATATTGT
beginning of TASY with 3Xflag F	TGACAAATCCTCTTCCACTGGT
213 BP TASY R	ATCACCCAAGGCGTTGAACA
TASY5	TCTGCTGGTCAAACTCACGT
Backbone R after Term	CAAGCGCGCAATTAACCCTC
(backbone1)_forward	TACAGACACTGGTCTGAACGTGGTATTGGTTGGGG

(backbone1)_reverse	GCGGCCAGCAAAACTAAGTTTCTTAGTATGATCCAATATCAAAGGAAATG
(URA17)_forward	TGGATCATACTAAGAAACTTAGTTTTGCTGGCCGCATCTTCTCAAATATGC
(ura17)_reverse	AGACGAAACTATATACGCAGGAAACGAAGATAAATCATGTCGAAA
(bakbone2)_forward	CGACATGATTTATCTTCGTTTCCTGCGTATATAGTTTCGTCTACCCTATGAACA
(Backbone2)_reverse	ATACCACGTTCAGACCAGTGTCTGTAGACGTAATCCAAAGCACC
(PArt1TASY)_forward	AAGCTCCCTCGTGCGCTCTCCTGTTCCGACC
(PArt1TASY)_reverse	AGAAATATCTTGACCGCAGTTTATATATATTTCAAGGATATACCATTCTAA TGT CTG
(part2TASY)_forward	CCTTGAAATATATATAAACTGCGGTCAAGATATTTCTTGAATCAGGCGC
(part2TASY)_reverse	GAACAGGAGAGCGCACGAGGGAGCTTCCAGGGGG
(Backbone1)_forward	GTTTTAGAGCTAGAAATAGCAAGTTAAAATAAGGC
(Backbone1)_reverse	GACATAACTAATTACATGACTCGAGAAGAGATCACACCTTCCT
(Backbone2)_forward	AGGTGTGATCTCTTCTCGAGTCATGTAATTAGTTATGTCACGCTTACGTTCACGCCC
(Backbone2)_(CAN1Y)_reverse	AGCCTTATTTTAACTTGCTATTTCTAGCTCTAAAACTCCTCCATAGAGAACGTATCAAAGTCCCATTCGCCACCCGAAGG
Gal1p F 3/4	GGTAATTAATCAGCGAAGCGATG
Gal1p F 1/2	CCCACAAACCTTCAAATCAACG
3xflag3	GGTTTGAATGACGTTATGATGCC

3Xflag4	CAAGGCGTTGAACATATCCTTG
DOWNTASY and UPCYC1 R	TCGGTTAGAGCGGATTCAAACT
Backbone after CYC1T R	CGC AAC GCA ATT AAT GTG AGT
(ORICYC1)_(KOZAK-SEQ)_reverse	CCGCAGACATTTTTTTGTTTGTTTATGTGTGTTTATTCGAAAC
GAL1 1/2 F	AGTAACCTGGCCCCACAAAC
Reverse3Xflag	ACCCAATTTAAGGCTTGTGGGA
(ORICYC1)_forward	GGTATATCCTTGAAATATATATAGGAGAAAATACCGCATCAGGAAATTG
BACKBONEtruncatedleu2d)_reverse	CCTGATGCGGTATTTTCTCCTATATATATTTCAAGGATATACCATTCTAATGTCTGCCCCTAAGAAGATCGTCG
CYC1T F Beginning	ATCCGCTCTAACCGAAAAGGAAGG
(vectorpart1)_reverse CASURA17	GATTTTGTTGATGCCATTATAGTTTTTTCTCCTTGACGTTAAAGTATAGAGG
(vectorpart1)_forward CAS URA17	GGTGTCTATTTTCTCTTCCATAAAAAAAGCCTGACTCCACTTCCCGC
vectorpart2_forward CASURA17	TTAATTTGCGGCCAAGCTTGATATCGAATTCCTGCAGCCCG
vectorpart2_reverse CASURA17	TGGAGTCAGGCTTTTTTTATGGAAGAGAAAATAGACACCAAAGTAGCC
term backbone kozak reverse	GAAAATCCTTGCTTAATCATCACCGAAACGCGCG
term backbone kozak forward	CCAATTCAAGTTTGAATCCGCTCTAACCGAAAAGGAAGG

TASYN50 kozak reverse	CCTTTTCGGTTAGAGCGGATTCAAACTTGAATTGGATCAATGTAGAC
TASYN50 kozak forward	CTGGTCCTGTCGTAATGTCCTCTTCCACTGGTACCTC
promoter backbone kozak reverse	GGAAGAGGACATTACGACAGGACCAGGACCACCACTTCCTCTCATAGACATTTTTTTGTTTGTTTATGTGTGTTTATTCGAAAC
promoter backbone kozak forward	CGTTTCGGTGATGATTAAGCAAGGATTTTCTTAACTTCTTCGGC
term backbone reverse	GAAAATCCTTGCTTAATCATCACCGAAACGCGCG
term backbone forward	CCAATTCAAGTTTGAATCCGCTCTAACCGAAAAGGAAGG
TASYN50 reverse	CCTTTTCGGTTAGAGCGGATTCAAACTTGAATTGGATCAATGTAGAC
TASYN50 forward	CTGGTCCTGTCGTAATGTCCTCTTCCACTGGTACCTC
promoter backbone reverse	GGAAGAGGACATTACGACAGGACCAGGACCACCACTTCCTCTCATTTTGTTTGTTTATGTGTGTTTATTCGAAAC
promoter backbone forward	CGTTTCGGTGATGATTAAGCAAGGATTTTCTTAACTTCTTCGGC
Beginning- TASY MBP primer F	TCCAGTTCGAGTTTATCATTATCAATACTGCCA
Beginning-TASY MBP primer R	GGTTTCAGAAACGACCTTAGAGGTACCAGTGGAAGAGG
End- TASY MBP primer F	GGTACCTCTAAGGTCGTTTCTGAAACCTCCTCTACCATCGTCG
End- TASY MBP primer R	GGCTTCTAATCCGTACTGGAGTTAGCATATCTACAATTGGGTGAAATGGGG
CRISPR Backbone 1 forward	GTTTTAGAGCTAGAAATAGCAAGTTAAAAT

CRISPR Backbone 1 reverse	TGGAGAATATACTAAGGGTACTGTTGACATTGCGAAGAGCGACAAAGATT
CRISPR Backbone 2 forward	CCGATAACAAAATCTTTGTCGCTCTTCGCAATGTCAACAGTACCCTTAGTATATTCTCCA
CRISPR Backbone 2 reverse	AAAGTCCCATTCGCCACCCGAA
(upstream)_reverse	CTAGCTCTAAAACTCTCCCGGGGGCGAGTCG
(upstream)_forward	CGTTTACAATTTCCTGATGCGGTATTTTCTCCTTACGC
F-up1622b GAL1	AACATTTAAGTCACAAGGAGGAATATCAGTT
R-up1622b	GCAGTATTGATAATGATAAACTCGAACTGAACTACTTTTCTTAAACTGTCAACAGCCA
F-dn1622b	TCACCCAATTGTAGATATGCTAACTCCGTAGATACTCGTCTTACGAAATTGGATATAGTT
R-dn1622b	ACTTTGGAAAAGAAGGTACGGACTACT
F-up1014a GAL1	TATTGACCAGTAGTCATATTACTGGCATATTATC
R-up1014a	GCAGTATTGATAATGATAAACTCGAACTGAGGATATTAATTTTAGGGTCTCTTGATGCAC
F-dn1014a	ACCCAATTGTAGATATGCTAACTCCCACTGTTTTCATCTAGACGTGGGAC
R-dn1014a	GCAATACCAGAGATGACTGGCC
F-TDH3-TASY-MBP-ADH1	CAGTTCGAGTTTATCATTATCAATACTGCC
R-TDH3-TASY-MBP-ADH1	GGAGTTAGCATATCTACAATTGGGTGAA
ADH1 fwd	CCCAAGACCATAAGCGAATTTCTTATG
ADH1 rev	GGAATTGTGAGCGGATAACGGAGTTAGCATATCTAC

1622bUP 8/10 F	TGCGAGCAGACTTTGTCCAT
1622bDOWN 2/10 R	TCGTTCTACAAGTCCAGCCA
1014a UP 8/10 F	AGGATTTCTATGTTCTCGAGGAGA
1014a DOWN 2/10 R	TCCGCCCTTTGCATCTATAAA
1014 (Scaffold)_forward	TTATGTGCGTATTGCTTTCAGTTTTAGAGCTAGAAATAGCAAGT TAAAATAAGGC
Scaffold_reverse	GCACCGACTCGGTGCCAC
Scaffold forward	GTTTTAGAGCTAGAAATAGCAAGTTAAAATAAGGC
(1622b)_(Scaffold)_forward	GTCACGTTCCTGAGGTTACTGTTTTAGAGCTAGAAATAGCAAGT TAAAATAAGGC

Table S3. **DNA sequences**

**Gene**	**Sequence**	**Reference**
E. coli MBP (Yeast codon-optimised)	TCTAAGATTGAAGAAGGTAAGTTGGTTATCTGGAT TAACGGTGACAAGGGTTACAACGGTTTGGCTGAAG TTGGTAAGAAATTTGAAAAAGATACCGGTATCAAG GTCACTGTTGAACACCCAGACAAGTTGGAAGAAA AGTTTCCACAAGTTGCTGCCACTGGTGATGGTCCA GACATTATCTTCTGGGCTCATGACAGATTCGGTGG TTACGCCCAATCCGGTTTGTTAGCCGAGATCACCC CAGATAAGGCTTTTCAAGATAAGTTGTATCCATTC ACTTGGGATGCCGTCAGATACAACGGTAAGTTAAT CGCCTACCCAATTGCTGTTGAAGCTTTGTCTTTGAT CTACAATAAGGACTTGTTACCTAACCCACCAAAGA	[1]

	CCTGGGAAGAAATCCCAGCTTTAGATAAGGAGTTAAAAGCTAAGGGTAAGTCCGCTTTGATGTTTAACTTGCAAGAACCATACTTCACTTGGCCATTGATCGCTGCTGATGGTGGTTACGCTTTTAAGTATGAAAACGGTAAATACGACATTAAGGATGTCGGTGTCGACAATGCTGGTGCTAAGGCCGGTTTAACTTTCTTAGTCGATTTGATTAAGAATAAACATATGAATGCTGACACTGATTACTCTATTGCTGAAGCTGCTTTCAACAAGGGTGAAACCGCTATGACTATTAACGGTCCATGGGCCTGGTCTAACATTGATACCTCTAAAGTCAACTACGGTGTCACCGTCTTGCCAACTTTTAAGGGTCAACCATCTAAGCCATTCGTCGGTGTCTTGTCTGCCGGTATTAACGCTGCCTCTCCAAATAAGGAATTGGCCAAGGAATTCTTAGAAAACTACTTGTTAACCGATGAAGGTTTAGAGGCCGTTAACAAGGATAAGCCATTAGGTGCTGTTGCTTTGAAGTCTTACGAAGAAGAGTTGGCTAAGGATCCAAGAATTGCTGCTACTATGGAAAACGCTCAAAAGGGTGAAATTATGCCAAACATCCCACAAATGTCTGCTTTCTGGTACGCTGTTCGTACCGCCGTCATTAATGCCGCTTCTGGTCGTCAAACTGTTGATGAAGCCTTGAAGGACGCTCAAACCAGAATTACTAAG	
Mutant variant of *Aequorea victoria GFP*	AGTAAAGGAGAAGAACTTTTCACTGGAGTTGTCCCAATTCTTGTTGAATTAGATGGTGATGTTAATGGGCACAAATTTTCTGTCAGTGGAGAGGGTGAAGGTGATGCAACATACGGAAAACTTACCCTTAAATTTATTTG	[2]

	CACTACTGGAAAACTACCTGTTCCATGGCCAACAC<br>TTGTCACTACTCTCACTTATGGTGTTCAATGCTTTT<br>CAAGATACCCAGATCAcATGAAACaGCATGACTTTT<br>TCAAGAGTGCCATGCCCGAAGGTTATGTACAGGAA<br>AGAACTATATTTTTCAAAGATGACGGGAACTACA<br>AGACACGTGCTGAAGTCAAGTTTGAAGGTGAT<br>ACCCTTGTTAATAGAATCGAGTTAAAAGGTAT<br>TGATTTTAAAGAAGATGGAAACATTCTTGGAC<br>ACAAATTGGAATACAACTATAACTCACACAAT<br>GTATACATCATGGCAGACAAACAAAAGAATG<br>GAATCAAAGcTAACTTCAAAATTAGACACAAC<br>ATTGAAGATGGAAGCGTTCAACTAGCAGACCA<br>TTATCAACAAAATACTCCAATTGGCGATGGCC<br>CTGTCCTTTTACCAGACAACCATTACCTGTCCA<br>CACAATCTGCCCTTTCGAAAGATCCCAACGAA<br>AAGAGAGACCACATGGTCCTTCTTGAGTTTGT<br>AACAGCTGCTGGGATTACACATGGCATGGATG<br>AACTATACAAA	
Yeast codon-optimised *TASY*	ATGTCCTCTTCCACTGGTACCTCTAAGGTCGTTTCT<br>GAAACCTCCTCTACCATCGTCGATGACATTCCAAG<br>ATTGTCTGCTAATTACCACGGTGACTTGTGGCATC<br>ATAACGTCATTCAAACCTTGGAAACTCCATTTAGA<br>GAATCTTCTACTTATCAAGAGAGAGCTGATGAATT<br>GGTTGTCAAGATCAAGGATATGTTCAACGCCTTGG	This Study

	GTGATGGTGATATCTCTCCATCTGCTTATGATACTG<br>CCTGGGTCGCTAGATTGGCTACCATCTCTTCCGAC<br>GGTTCCGAAAAGCCAAGATTCCCACAAGCCTTAAA<br>TTGGGTTTTTAACAACCAATTGCAAGACGGTTCTT<br>GGGGTATTGAATCTCATTTCTCTTTGTGTGATAGAT<br>TGTTGAACACCACTAACTCCGTCATTGCCTTGTCTG<br>TTTGGAAGACTGGTCACTCTCAAGTTCAACAAGGT<br>GCCGAATTCATTGCCGAAAACTTGAGATTATTGAA<br>CGAAGAAGATGAATTGTCTCCAGACTTCCAAATCA<br>TTTTTCCAGCTTTGTTGCAAAAGGCCAAGGCCTTA<br>GGTATCAACTTGCCATACGACTTGCCATTCATCAA<br>GTACTTGTCTACTACCAGAGAAGCTAGATTGACTG<br>ACGTCTCCGCTGCTGCTGACAACATTCCAGCCAAC<br>ATGTTGAATGCCTTGGAAGGTTTAGAAGAAGTCAT<br>TGATTGGAACAAGATTATGAGATTCCAATCTAAAG<br>ACGGTTCTTTTTTGTCTTCCCCTGCTTCTACTGCTTG<br>TGTCTTGATGAACACCGGTGATGAGAAGTGTTTCA<br>CTTTCTTGAATAACTTGTTGGATAAATTCGGTGGTT<br>GTGTTCCATGTATGTATTCCATTGATTTATTGGAAA<br>GATTGTCTTTAGTTGACAATATTGAACATTTGGGT<br>ATCGGTAGACACTTCAAGCAAGAAATTAAGGGTG<br>CTTTGGATTACGTCTACAGACACTGGTCTGAACGT<br>GGTATTGGTTGGGGTAGAGATTCTTTGGTTCCAGA<br>TTTAAACACTACTGCCTTGGGTTTGCGTACCTTGAG<br>AATGCACGGTTACAACGTTTCTTCCGACGTTTTGA	

	ACAACTTCAAGGATGAAAACGGTAGATTCTTTTCC<br>TCTGCTGGTCAAACTCACGTCGAATTAAGATCCGT<br>TGTCAACTTGTTCAGAGCTTCTGATTTGGCCTTCCC<br>AGACGAAAGAGCTATGGATGATGCTAGAAAATTC<br>GCTGAACCATATTTGAGAGAGGCCTTGGCCACCAA<br>GATTTCTACCAACACTAAGTTGTTCAAGGAGATTG<br>AATACGTTGTCGAATACCCATGGCACATGTCCATC<br>CCAAGATTGGAAGCTAGATCCTATATTGACTCCTA<br>CGACGATAACTACGTTTGGCAAAGAAAAACCTTGT<br>ACCGTATGCCATCCTTGTCTAACTCCAAGTGTTTGG<br>AGTTAGCTAAATTAGACTTCAATATCGTCCAATCC<br>TTACATCAAGAAGAATTGAAATTGTTGACCAGATG<br>GTGGAAGGAATCTGGTATGGCTGATATCAACTTCA<br>CCAGACACAGAGTCGCCGAAGTTTACTTCTCCTCT<br>GCTACTTTCGAACCAGAATACTCCGCTACCAGAAT<br>TGCTTTCACTAAGATCGGTTGTTTACAAGTTTTATT<br>CGATGATATGGCTGACATTTTCGCTACTTTGGATG<br>AATTGAAGTCTTTCACTGAAGGTGTTAAGAGATGG<br>GATACTTCTTTGTTGCATGAAATCCCAGAATGTAT<br>GCAAACCTGTTTTAAGGTTTGGTTTAAGTTGATGG<br>AGGAAGTCAACAACGACGTTGTTAAGGTTCAAGGT<br>AGAGACATGTTGGCCCACATCAGAAAGCCATGGG<br>AATTGTACTTCAACTGTTACGTCCAAGAAAGAGAA<br>TGGTTGGAAGCTGGTTATATCCCTACCTTCGAAGA<br>ATACTTGAAGACTTACGCTATCTCTGTCGGTTTAG	

	GTCCTTGTACTTTGCAACCAATTTTGTTGATGGGTG AATTAGTTAAGGACGATGTTGTTGAAAAAGTTCAC TACCCATCCAACATGTTCGAATTAGTTTCCTTGTCC TGGAGATTAACCAATGACACCAAGACTTACCAAGC CGAAAAGGCTAGAGGTCAACAAGCTTCTGGTATCG CTTGTTACATGAAGGATAATCCAGGTGCCACTGAA GAAGATGCTATTAAGCACATTTGTAGAGTTGTTGA CAGAGCTTTGAAGGAAGCCTCTTTCGAATACTTTA AGCCATCCAACGATATCCCAATGGGTTGCAAGTCT TTCATCTTCAACTTGAGATTGTGTGTCCAAATCTTC TACAAGTTTATCGACGGTTACGGTATTGCTAACGA AGAAATCAAGGATTACATTAGAAAAGTCTACATTG ATCCAATTCAAGTTTGA	

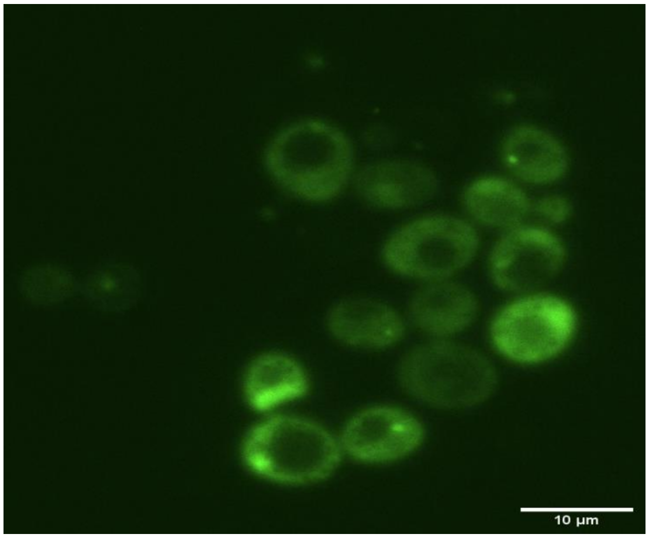

Figure S1. **Fluorescence imaging of GFP-tagged *TASY* showing spotted subcellular localization consistent with poor TASY solubility**

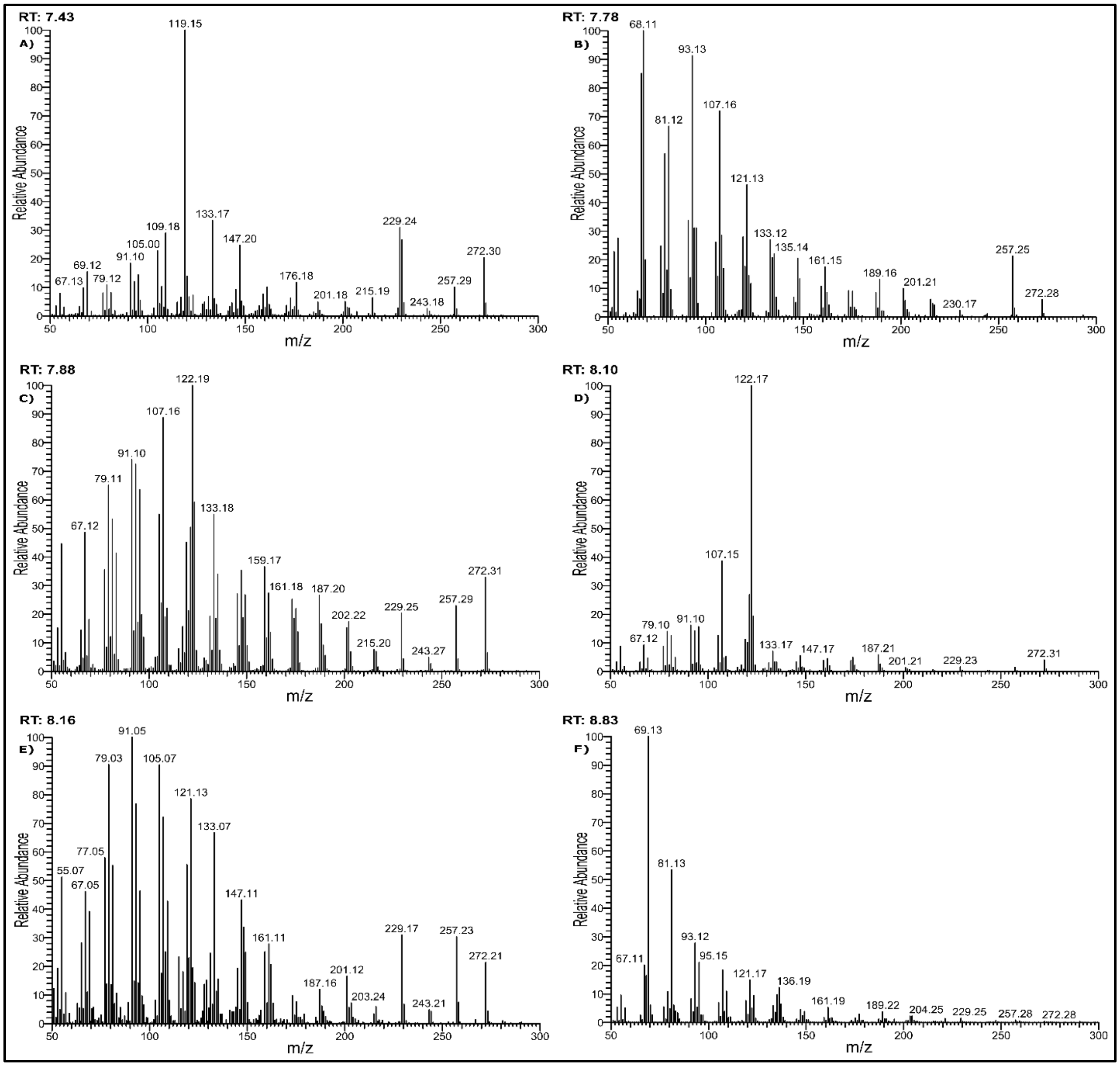

Figure S2. **Mass spectra for compounds produced by LRS5**. A) Verticillene; B) Diterpene 1; C) Iso-taxadiene; D) Taxadiene; E) Diterpene 2; F) GGOH

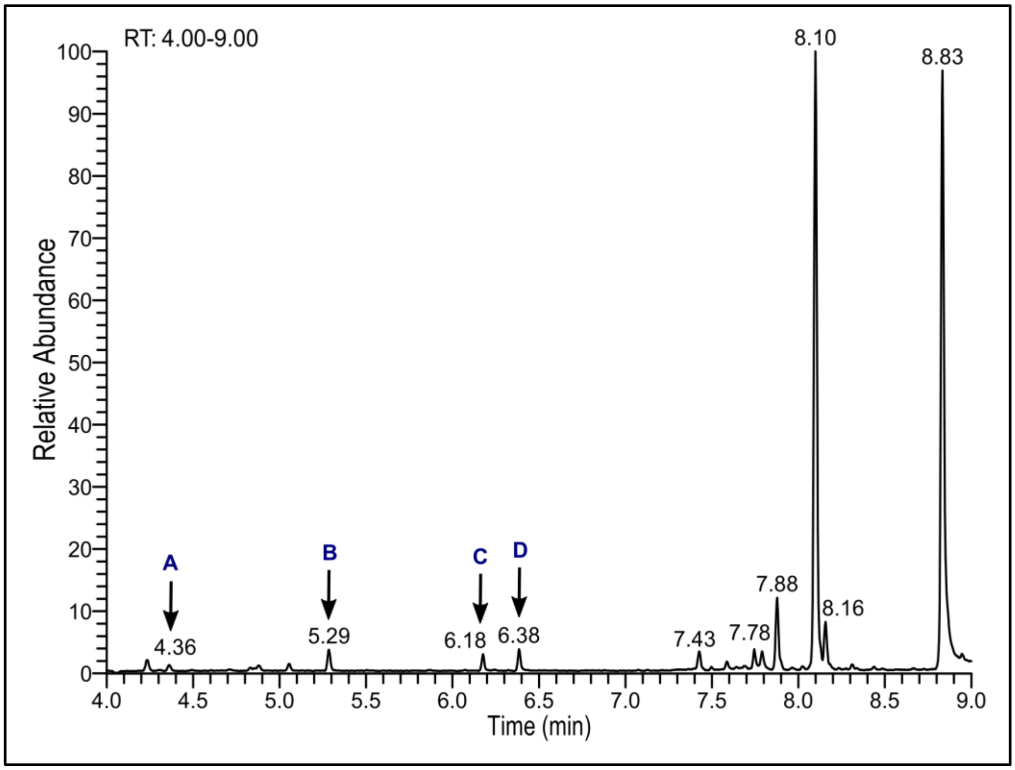

Figure S3. **LRS5 gas chromatogram showing additional potential terpenoids.** Compounds produced during LRS5 shake flask cultivation at 30 °C. The mass spectra of unknown potential terpenoids A, B, C and D are shown in Figure S4.

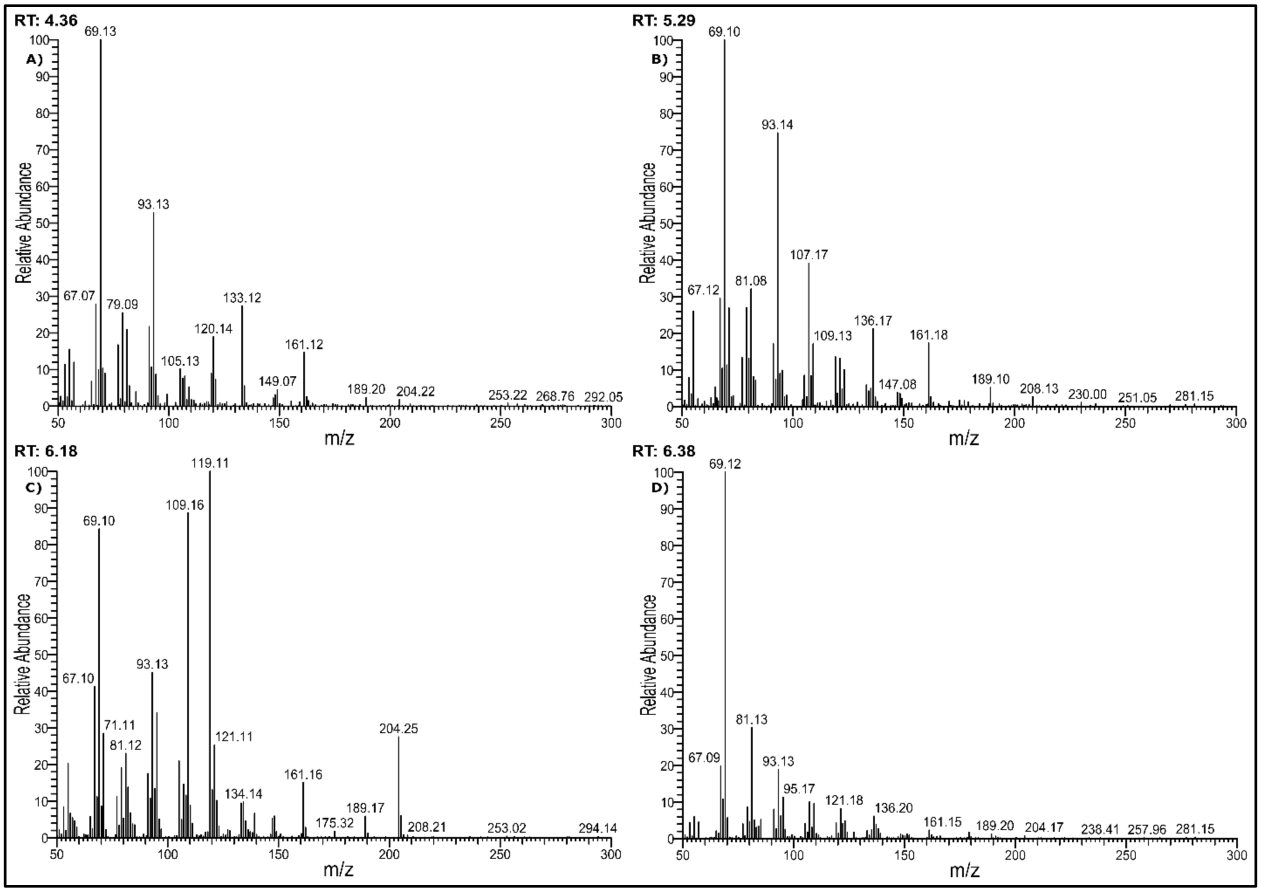

Figure S4. **Mass spectra of the LRS5 additional potential terpenoids (A-D) as shown in Figure S3.**

References

1. Sun P, Tropea JE, Waugh DS. Enhancing the solubility of recombinant proteins in *Escherichia coli* by using hexahistidine-tagged maltose-binding protein as a fusion partner. Methods Mol Biol. 2011;705:259-274.

2. Houser JR, Ford E, Chatterjea SM, Maleri S, Elston TC, Errede B. An improved short-lived fluorescent protein transcriptional reporter for *Saccharomyces cerevisiae*. Yeast. 2012;29:519–530.

3. Schneider CA, Rasband WS, Eliceiri KW. NIH Image to ImageJ: 25 years of image analysis. Nat Methods. 2012;9:671–675.
